# A prospective observational cohort study of the efficacy of tofacitinib plus iguratimod on rheumatoid arthritis with usual interstitial pneumonia

**DOI:** 10.3389/fimmu.2023.1215450

**Published:** 2023-08-23

**Authors:** Shuhua Wang, Yao Li, Yanchun Tang, Weilin Xie, Yue Zhang, Qingyan Liu

**Affiliations:** Department of Rheumatology, the Qingdao University Medical College Affiliated Yantai Yuhuangding Hospital, Yantai, China

**Keywords:** rheumatoid arthritis with usual interstitial pneumonia, tofacitinib plus iguratimod, conventional synthetic disease-modifying anti-rheumatic drugs, dual treat-to-target, efficacy

## Abstract

**Objectives:**

This study aims to assess the efficacy of tofacitinib (TOF) plus iguratimod (IGU) in rheumatoid arthritis (RA) with usual interstitial pneumonia (UIP) (RA-UIP).

**Methods:**

This was a prospective observational cohort, single-center study. Data from 78 RA-UIP patients treated with TOF plus IGU, IGU plus conventional synthetic disease-modifying anti-rheumatic drugs (csDMARDs), and csDMARDs were analyzed. Clinically relevant responses in RA activity assessment, pulmonary function tests (PFTs), and high-resolution computed tomography (HRCT) assessment at baseline and follow-up were compared between groups to evaluate the efficacy of TOF plus IGU.

**Results:**

A total of 78 patients were followed up for at least 6 months after treatment. There were significant changes in sedimentation rate (ESR), C reactive protein (CRP), and disease activity score (DAS) 28-CRP during the follow-up within each treatment group, but there was no statistically significant difference between the two groups. After 6 months of TOF plus IGU treatment, forced vital capacity (FVC)% (84.7 ± 14.7 vs. 90.7 ± 15.4) and HRCT fibrosis score (7.3 ± 3.4 vs. 7.0 ± 5.6) showed a significant improvement compared to the csDMARDs group (*P* = 0.031, *P* = 0.015). The TOF plus IGU-treated patients had a significantly higher regression and lower deterioration than the csDMARDs-treated patients (*P* = 0.026, *P* = 0.026) and had a significantly higher response (regression + stability), with overall response rates of 66.7% (16/24) vs. 35.7% (10/28) (*P* = 0.027), respectively.

**Conclusion:**

Our results indicate that TOF plus IGU can simultaneously relieve RA and RA-UIP and be better than the csDMARDs with a higher response rate in RA-UIP, which may be a potential choice for “dual treat-to-target”.

## Introduction

Rheumatoid arthritis (RA), a chronic systemic inflammatory and autoimmune disease, features articular and extra-articular manifestations. Interstitial long disease (ILD) is a common extra-articular manifestation of RA and was reported to be up to 30% (1). RA-ILD is the second leading cause of mortality, just behind cardiovascular disease (2).

RA-ILD has a variety of subtypes in radiography and histopathology. Usual interstitial pneumonia (UIP), accounting for 40%–60% of patients with RA-ILD, is the most frequent imaging pattern, followed by nonspecific interstitial pneumonia at approximately 40%. Unclassified patterns are estimated in up to 6% of RA patients (3). Remarkably, the UIP pattern confers a poorer prognosis, with survival rates paralleling those seen in idiopathic pulmonary fibrosis (IPF) (4). A 1.6-fold-higher risk of death for those with a UIP pattern was observed in a meta-analysis of 10 cohort studies, including 1,256 patients with RA-ILD, compared with other patterns (5).

RA-UIP shares similarities with IPF in radiology and histopathology, indicating the necessity of saving RA-UIP. However, how to reverse or stabilize the lesions remains challenging. The therapeutic choices in RA-ILD are complicated by many conventional synthetic disease-modifying anti-rheumatic drugs (csDMARDs) due to possible pulmonary toxicity and unclear efficacy on pulmonary involvement (6). csDMARDs, including methotrexate (MTX), leflunomide (LEF), anti-tumor necrosis factor-α agents, rituximab, and tocilizumab or even abatacept, have been frequently reported to induce and worsen the existing RA-ILD (2).

Tofacitinib (TOF), approved to control RA, is a small-molecule Janus kinase (JAK)1/JAK3 and partly JAK2/tyrosine kinase 2 inhibitor. JAK/signal transducer and activator of transcription pathway have been involved in pulmonary fibrosis (7, 8). Recently, *in vivo* and *in vitro* studies were investigating TOF in ILDs. The results collectively indicated that TOF, which slows down the progression of connective tissue disease-ILD, might be an effective therapeutic strategy for RA-UIP (9–12).

IGU with novel anti-inflammatory and immunomodulatory properties was developed as a new anti-rheumatic drug and has been widely used for the treatment of RA. Moreover, research had also shown that IGU could have potential anti-fibrotic properties in pulmonary fibrosis (13–15).

No additional studies have been reported on the efficacy of TOF plus IGU in RA-UIP. Therefore, we conducted this observational study to collect the clinical data of 78 RA-UIP patients treated with TOF plus IGU, IGU plus csDMARDs, or csDMARDs and compared the treatment responses between groups to better understand their safety and efficacy against RA-UIP.

## Methods

### Study design and participants

We conducted the prospective observational, single-center study in RA-UIP patients who were registered in the Department of Rheumatology of Yantai Yuhuangding Hospital from July 2020 to July 2022. The RA-UIP patients enrolled by my medical team were treated with TOF plus IGU, or IGU plus csDMARDs if suitable, while the RA-UIP patients enrolled and treated suitably with csDMARDs without JAK inhibitors, IGU, or biologic DMARDs were selected as the control group. All patients were followed up for at least 6 months.

By having the questionnaires filled in, we obtained the demographic data, clinical characteristics, and laboratory indicators at baseline.

### Inclusion criteria

Patients aged over 18 years old were included and met the American College of Rheumatology or European League Against Rheumatism classification criteria for RA diagnosis (16) and the American Thoracic Society or the European Respiratory Society 2002 criteria for UIP diagnosis concomitantly (17).

### Exclusion criteria

Pulmonary interstitial disease caused by pneumoconiosis, inhaled organic matter, cardiac insufficiency, and other connective tissue diseases were excluded.

### Ethical considerations

This study was approved by Yantai Yuhuangding Hospital of Qingdao University Ethics Committee (approval number: 2022-014, Yantai, China). All patients signed the informed consent form.

### Treatment regimens

During the study, the TOF plus IGU-treated patients were treated with TOF 5 mg twice daily plus IGU 25 mg twice daily. The IGU plus csDMARDs-treated patients were treated with IGU 25 mg twice daily plus one of MTX, LEF, hydroxychloroquine, sulfasalazine, and tripterygium glycosides, and the csDMARDs-treated patients were treated with MTX or LEF plus one of hydroxychloroquine, sulfasalazine, and tripterygium glycosides. All the patients in the three groups were given ≤15 mg daily prednisone or equivalent. In addition, any previously prescribed glucocorticoids or csDMARDs could remain unchanged, be reduced, or discontinued, but no other glucocorticoids, csDMARDs or biologic agents, were permitted to be added.

### Evaluation protocol

The treatment response of patients at 6 months after treatment was evaluated using the collected patient information. A clinically relevant response was defined as changes in RA activity assessment [erythrocyte sedimentation rate (ESR), C reactive protein (CRP), and disease activity score (DAS) 28-CRP], PFTs [forced vital capacity (FVC)%, and carbon monoxide diffusion capacity single-breath method (DLCO SB%)], and HRCT scores [total ground-glass opacity (GGO)/fibrosis score] at baseline and follow-up. The clinical indicators were compared between the three groups to evaluate the efficacy of TOF plus IGU. At each follow-up, episodes of signs of infection such as fever, sore throat, cough, or diarrhea were recorded.

According to the chest HRCT results performed at baseline and follow-up, GGO and fibrosis were classified and scored to evaluate the HRCT findings (18). HR CT assessment categorized the UIP course as regression, stability, or deterioration. Deterioration and regression were defined by an increase or decrease of at least 10% of the overall disease extent, respectively, while stability was defined by changes of less than 10% (19).

Two experienced radiologists blinded to the RA-UIP patients’ conditions independently assessed the HRCT images. Divergent conclusions were resolved by a consensus between the two observers.

### Adverse events

Adverse reactions related to the use of TOF were evaluated, including infection, lipid changes, creatinine clearance reduction, liver enzyme level increase, and blood routine changes.

### Statistical analysis

Data were processed with SPSS version 23.0 (IBM, Armonk, NY, USA). Normal distribution was presented with the mean and standard deviation and tested by *t*-test. Abnormal distribution was presented with the median and interquartile ranges and tested by nonparametric test. The counting data were presented (%), and the comparison between the two groups made use of χ^2^ test. With the two-sided coupled *t*-test, values at baseline and follow-up were compared, and *P <*0.05 was considered statistically significant.

## Results

### Patients’ characteristics

In total, 84 patients were enrolled: six patients were lost to follow-up, and 78 patients were ultimately followed up. A total of 24 RA-UIP patients (12 female and 12 male) were treated with TOF plus IGU, 26 RA-UIP patients (12 female and 14 male) were treated with IGU plus csDMARDs, and 28 RA-UIP patients (12 female and 16 male) were treated with csDMARDs as the control group. All the treatment groups were followed up for at least 6 months, with a mean disease duration of 15.1 ± 9.6, 14.5 ± 12.5, and 14.2 ± 11.4 months (*P* = 0.519), respectively. No differences were observed in the demographic and clinical features between the three groups. For the follow-up time, there was no statistical differences among the three groups (*P* = 0.881). For the treatments in combination with medium or low dose of glucocorticoids, there was no significant difference at baseline and follow-up data between the three groups (*P* = 0.276) (**Table 1**).

**Table 1:** Demographic and clinical characteristics of the RA-UIP patients (*n* = 78).

Variables	TOF + IGU *n* = 24	IGU + csDMARDs *n* = 26	csDMARDs *n* = 28	*p*-value
Male sex	12 (50.0)	14 (53.8)	12 (42.9)	0.785
Age, years	64.2 ± 6.8	61.9 ± 10.2	63.5 ± 8.1	0.542
Disease duration for RA, months	15.1 ± 9.6	14.5 ± 12.5	14.2 ± 11.4	0.519
Follow-up time, months	5.9 ± 3.6	6.5 ± 3.5	6.2 ± 4.4	0.881
Smoking status, yes	4 (16.7)	5 (19.2)	5 (17.9)	0.832
RF positive	20 (83.3)	23 (88.4)	24 (85.7)	0.580
Anti-CCP positive	21 (87.5)	21 (80.8)	23 (82.1)	0.552
Presence of comorbidity	22 (91.7)	24 (92.3)	23 (82.1)	0.227
Steroids, csDMARDs used at baseline				
Steroids	6 (23.1)	5 (19.5)	8 (28.6)	0.552
MTX	2 (7.7)	3 (11.5)	5 (17.9)	0.324
LEF	3 (11.5)	4 (15.4)	3 (10.7)	0.997
Hydroxychloroquine	3 (11.5)	2 (7.7)	4 (14.3)	0.441
Sulfasalazine	1 (3.8)	4 (15.4)	2 (7.1)	0.181
Tripterygium glycosides	4 (15.4)	5 (19.2)	7 (25.0)	0.611
Steroids, csDMARDs used at follow-up				
Steroids	17 (70.8)	18 (69.2)	16 (57.1)	0.358
MTX	–	11 (42.3)	17 (60.7)	0.387
LEF	–	6 (23.1)	11 (39.3)	0.200
Hydroxychloroquine	–	4 (15.4)	7 (25.0)	0.380
Sulfasalazine	–	2 (7.7)	11 (39.3)	0.007
Tripterygium glycosides	–	3 (11.5)	10 (35.7)	0.063

Data are presented as number (percentage, %) and means and standard deviations where appropriate.

RF, rheumatoid factor; CCP, anti-cyclic citrullinated peptide; csDMARDs, conventional synthetic disease-modifying anti-rheumatic drugs; MTX, methotrexate; LEF, leflunomide.

### Disease activity and remission

A total of 78 patients were followed up for at least 6 months after treatment. There were significant changes in CRP, ESR and DAS28-CRP during the follow-up within each treatment group, but there was no statistically significant difference between the two groups. The decrease of DAS28-CRP in the TOF plus IGU group (4.8 ± 1.6 vs. 3.8 ± 1.9) was greater than that in the csDMARDs group (4.3 ± 2.2 vs. 3.5 ± 1.8), but statistical significance was not detected (*P* = 0.162).

The TOF plus IGU group showed a slight progress in HRCT GGO scores (2.1 ± 0.9 vs. 3.4 ± 6.7) at 6 months of follow-up, and there was no statistically significant difference compared to the csDMARDs group *(P* = 0.359). After 6 months of TOF plus IGU treatment, FVC% (84.7 ± 14.7 vs. 90.7 ± 15.4) and HRCT fibrosis score (7.3 ± 3.4 vs. 7.0 ± 5.6) showed a significant improvement, with significant statistical differences compared to the csDMARDs group (*P* = 0.031, *P* = 0.015).

There was a slight decrease in FVC% and DLCO SB% of the IGU plus csDMARDs group and the csDMARDs group compared to baseline, but there was no statistically significant difference between the two groups (*P* = 0.325, *P* = 0.471). Compared to baseline, the HRCT GGO scores and fibrosis scores of the IGU plus csDMARDs group and the csDMARDs group both had a slight change, with no statistically significant difference between the two groups (*P* = 0.576, *P* = 0.846).

In the TOF plus IGU group, eight patients (8/24, 33.3%) showed RA-UIP deterioration, six patients (6/24, 25.0%) were considered stable, and 10 patients (10/24, 41.7%) demonstrated RA-UIP regression. In the IGU plus csDMARDs group, 14 patients (14/26, 53.8%) showed RA-UIP deterioration, eight patients (8/26, 30.8%) were stable, and four patients (4/26, 15.4%) demonstrated RA-UIP regression. In the csDMARDs group, 18 patients (18/28, 64.3%) showed RA-UIP deterioration, six patients (6/28, 21.4%) were stable, and four patients (4/28, 14.3%) demonstrated RA-UIP regression. The TOF plus IGU-treated patients had a significantly higher regression and lower deterioration than the csDMARDs-treated patients (*P* = 0.026, *P* = 0.026).

The IGU plus csDMARDs-treated patients had a higher response (regression + stability, 46.2%, 12/26) than the csDMARDs-treated patients (35.7%, 10/28), but there was no statistical significance (*P* = 0.435). The TOF plus IGU-treated patients had a significantly higher response than the csDMARDs-treated patients, with overall response rates of 66.7% (16/24) vs. 35.7% (10/28) (*P* = 0.027), respectively (**Table 2**).

**Table 2:** Comparison of clinical indices, PFTs and HRCT scores of patients with RA-UIP at baseline and follow-up.

Variables	TOF plus IGU *n* = 24			IGU + csDMARDs *n* = 26			csDMARDs *n* = 28	
	Baseline	Follow-up	*p*-value	Baseline	Follow-up	*p*-value	Baseline	Follow-up
RF (<20 IU/mL)	63.2 ± 60.5	57.6 ± 53.9	0.756	65.5 ± 37.2	60.4 ± 32.6	0.564	76.9 ± 58.6	69.5 ± 46.2
CCP (<5 RU/mL)	141.5 ± 64.6	136.5 ± 74.5	0.342	196.6 ± 8.5	152.0 ± 56.4	0.897	140.0 ± 80.4	130.5 ± 77.2
CRP (<10 mg/L)	28.2 ± 56.7	20.7 ± 24.8	0.563	31.9 ± 3.3	23.2 ± 35.2	0.247	28.3 ± 31.1	23.7 ± 16.6
ESR (<15 mm/h)	41.2 ± 21.7	30.3 ± 18.2	0.786	37.7 ± 26.7	34.3 ± 20.4	0.768	28.6 ± 14.5	29.3 ± 23.1
DAS28-CRP	4.8 ± 1.6	3.8 ± 1.9	0.162	5.4 ± 1.3	4.0 ± 1.7	0.426	4.3 ± 2.2	3.5 ± 1.8
FVC%	84.7 ± 14.7	90.7 ± 15.4	0.031	82.9 ± 7.3	77.4 ± 11.8	0.325	81.6 ± 11.2	75.9 ± 6.8
DLCO SB%	69.4 ± 16.1	61.6 ± 14.6	0.353	63.5 ± 19.5	57.8 ± 20.1	0.471	54.5 ± 11.3	49.4 ± 24.0
GGO score	2.1 ± 0.9	3.4 ± 6.7	0.359	1.62 ± 1.1	1.7 ± 0.9	0.576	2.3 ± 1.3	2.1 ± 1.4
Fibrosis score	7.3 ± 3.4	7.0 ± 5.6	0.015	7.8 ± 3.9	8.9 ± 4.1	0.846	6.4 ± 3.9	7.9 ± 5.2
Fibrosis score R	–	10 (41.7)	0.026	–	4 (15.4)	0.909	–	4 (14.3)
Fibrosis score S	–	6 (25.0)	0.761	–	8 (30.8)	0.434	–	6 (21.4)
Fibrosis score D	–	8 (33.3)	0.026	–	14 (53.8)	0.435	–	18 (64.3)
Fibrosis score R + S	–	16 (66.7)	0.027	–	12 (46.2)	0.435	–	10 (35.7)

Data are presented as means and standard deviations or number (percentage, %) where appropriate.

RF, rheumatoid factor; CCP, anti-cyclic citrullinated peptide; CRP, C reactive protein; ESR, erythrocyte sedimentation rate; DAS, Disease Activity Score; FVC, forced vital capacity; DLCO SB, carbon monoxidediffusion capacity single-breath method; GGO, ground glass opacity; R, regression; S, stability; D, deterioration.

We closely monitored TOF- or IGU-related side effects during the follow-up. At 3 weeks after using TOF, one patient developed herpes zoster. No patient discontinued TOF or IGU due to side effects or poor efficacy. All patients reduced the doses of steroids.

## Discussion

Fibrotic diseases, including RA-UIP, tend to be less responsive to steroids and most csDMARDs. The course of RA-UIP resembles that of IPF. In some cases, RA-UIP has been reported to progress quickly and have a fatal course despite strong immunosuppressive treatments such as MTX, LEF, and even cyclophosphamide and pulse therapies. Consequently, new treatment strategies are needed.

**Figure 1:**
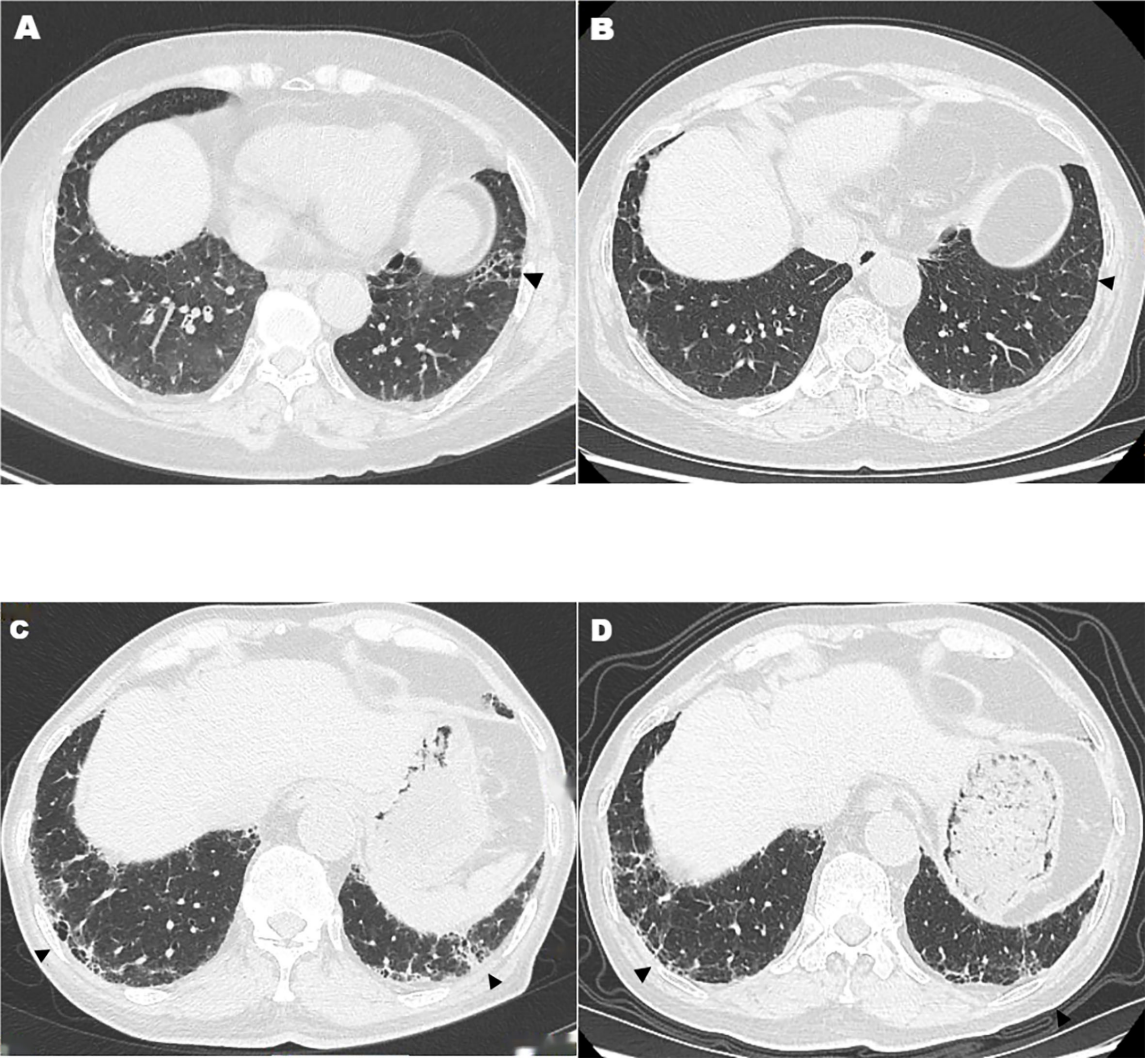
High-resolution computed tomography features after tofacitinib plus iguratimod treatment. Arrowheads show decreased fibrotic lesions that were partly absorbed, attenuated, or evolved into ground-glass opacity **(A–D)**.

This may be the first and largest prospective observational, single-center study to assess the efficacy and safety of TOF plus IGU in a homogeneous RA-UIP patient cohort. Our study showed that, in the control of RA-articular manifestations, TOF plus IGU is equivalent to IGU plus csDMARDs, and csDMARDs was same as that of GGO in RA-UIP. The GGO scores in TOF plus IGU-treated patients were increased. These changes in HRCT were not considered as deteriorating because it might be due to the fact that the fibrotic lesions were partly absorbed, attenuated, and evolved into GGO (**Figure 1**). When TOF plus IGU was given, the HRCT fibrosis scores and FVC% were improved, even significantly reversed in as few as 6 months with a higher response rate, better than csDMARDs. Nevertheless, this still needs more studies to verify the results considering that the TOF plus IGU group had a small number of patients. This combined strategy realizes that both RA and RA-UIP can be relieved simultaneously and maybe a potential choice for “dual treat-to-target” due to the combined anti-inflammatory and anti-fibrotic properties of JAKis and IGU (20).

Some preliminary data have investigated the combined anti-inflammatory and anti-fibrotic properties of JAKis and IGU in ILD or RA-ILD (9–15). When TOF and IGU were given in combination, anti-fibrosis effects were shown in HRCT findings and might slow down fibrosis in RA-UIP. Similar findings were also found in a retrospective study recently. The study by Tardella M et al. highlighted the efficacy of TOF and abatacept in RA-ILD patients, and they concluded that JAKis are effective in slowing down fibrosis in RA-ILD and should be considered as a first-choice therapy in RA patients with active synovitis and ILD before a stage of extensive fibrosis (12). Shu P et al. observed that IGU plus glucocorticoid or cyclophosphamide markedly increased the lung function indexes compared to glucocorticoid or cyclophosphamide in RA-ILD patients (21). Anyway, data on the efficacy of TOF and IGU in the treatment of RA-ILD are still limited to sporadic case reports or studies with small groups of patients and have been hardly reported.

Nintedanib and pirfenidone were approved in chronic fibrosing progressive ILD, including RA-ILD. Definitely, as complementary remedy to DMARDs, anti-fibrotic drugs might offer an important therapeutic choice in progressive RA-UIP. However, besides the high price weakening the adherence to therapy, it could also result in additional side effects, such as diarrhea, liver toxicity, and so on. Therefore, it may be advisable to choose drugs that could be effective against both the articular and extra-articular manifestations in RA, and TOF plus IGU might be the best choice.

After adding TOF plus IGU, the improvement of the UIP in RA patients in our study may mainly result from the effect of TOF, but it may also be the synergy effect of the two combined drugs. *In vivo* and *in vitro* studies require being accumulated in the future to verify if there was some synergy existing in the two drugs and clarify these synergistic anti-fibrosis effects exerted by the combined therapy. Either way, our results suggest that TOF plus IGU is effective against RA-UIP and might delay the intervention of nintedanib and pirfenidone.

In literature, the most widely used method of assessing pulmonary fibrosis is the trend of PFT parameters, especially FVC and DLCO. Both pulmonary and extra-pulmonary factors could influence the FVC and DLCO. HRCT, with its outstanding advantages, can better reflect the therapeutic effect demonstrated by TOF plus IGU, particularly the quickness of estimating the fibrosis percentage. Two different methods were taken to assess the response to treatment in our study. This aspect may be a distinctive feature of our research.

This study had some limitations. In this pilot, single-center study enrolling small groups of patients with RA-UIP and with a relatively short follow-up period, it is difficult to obtain any conclusion on long-term efficacy. Furthermore, this is a combined therapy, so it is uncertain whether there is a synergistic effect existing in the two drugs, and the effectiveness contributed by each drug cannot be evaluated precisely. In addition, lung fibrosis in the patients is mild to moderate, with less than 15 fibrosis scores, so our findings may not be generalized to patients with severe subtypes of RA-UIP, which can lead to respiratory failure or death.

## Conclusion

In conclusion, our results indicate that TOF combined with IGU can simultaneously relieve RA and RA-UIP and be better than csDMARDs, with a higher response rate in RA-UIP patients. We believe that our results may shed light on the therapy of RA-UIP, and TOF plus IGU may be a potential choice for “dual treat-to-target” in RA. In addition, as most csDMARDs are usually ineffective for RA-UIP, the combined strategy could be used in most cases, especially where csDMARDs were poorly tolerated or insufficiently effective. Prospective studies with a larger cohort may be inspired to verify whether this finding applies to other cases and clarify these anti-fibrosis effects exerted by the combined therapy in the future.

## Data availability statement

The original contributions presented in the study are included in the article/supplementary material. Further inquiries can be directed to the corresponding author.

## Ethics statement

The studies involving humans were approved by Yantai Yuhuangding Hospital Ethics Committee (approval number 2022-014). The studies were conducted in accordance with the local legislation and institutional requirements. The participants provided their written informed consent to participate in this study. Written informed consent was obtained from the individual(s) for the publication of any potentially identifiable images or data included in this article.

## Author contributions

All authors were involved in study management. WX: conception, design, preparation of the manuscript, review of criteria, and final approval of the manuscript. SW: conception, design, and preparation of the manuscript. YL: conception, design, and preparation of the manuscript. YT, YZ, and QL: conception, design, and preparation of the manuscript. All authors contributed to the article and approved the submitted version.
